# Internalising and externalising in early adolescence predict later executive function, not the other way around: a cross-lagged panel analysis

**DOI:** 10.1080/02699931.2021.1918644

**Published:** 2021-04-26

**Authors:** Georgina Donati, Emma Meaburn, Iroise Dumontheil

**Affiliations:** a Centre for Brain and Cognitive Development, Department of Psychological Sciences, Birkbeck, University of London, London, UK; b Child and Adolescent Psychiatry, University of Oxford, Warneford Hospital, Oxford, UK

**Keywords:** Adolescence, internalising, externalising, executive function, ALSPAC

## Abstract

Developmental changes in the brain networks involved in emotion regulation are thought to contribute to vulnerability to mental health problems during adolescence. Executive control is often viewed as allowing top-down regulation of emotional responses. However, while associations between executive control and mental health are commonly observed in both clinical and non-clinical populations, the direction of these associations remains unclear. Low, or immature, cognitive control could limit emotion regulation. Reversely, high emotionality could impede cognitive functioning. The scarcity of longitudinal studies testing for bi-directional effects, particularly in adolescence, has made it difficult to draw conclusions. This study analysed data from 1,445 participants of a longitudinal cohort in a cross-lagged panel design to understand bi-directional longitudinal associations between executive function and emotional behaviours across adolescence. Executive function was assessed using experimental working memory and inhibitory control tasks, emotional behaviours through parental report of internalising and externalising behaviours. Cross-sectional associations were replicated. Controlling for cross-sectional associations, early executive functions were not found to predict later emotional behaviours. Instead, early emotional behaviours predicted later executive function, with the strongest link observed between early externalising and later working memory. These results suggest that emotional well-being may affect the maturation of executive function during adolescence.

Many mental health conditions have an age of onset during adolescence (Giedd et al., 2008; Rapee et al., 2019), coinciding with substantial changes occurring in brain areas governing cognitive control and emotional reactivity (Crone & Dahl, 2012). This has led to a hypothesis that relatively poorer cognitive control, combined with increasingly reactive subcortical regions, make emotion regulation difficult for adolescents, putting them at risk for developing mental health issues (Crone & Dahl, 2012). Studies in adults have shown associations between activity in the prefrontal cortex (PFC), down-regulation of amygdala activity, and better mental well-being (Frank et al., 2014). These studies provide a mechanistic understanding for findings that individual differences in cognitive control, or executive function (EF), have been associated with internalising and externalising behaviours. Internalising behaviours are directed inwards and include fearfulness, social withdrawal and anxiety, while externalising behaviours are directed outwards and include physical aggression, disobedience and substance abuse (American Psychiatric Association, 2013). Poor EF has therefore been proposed as a risk factor for developing psychopathologies (Snyder et al., 2015). However, there is also research showing that emotion can interfere with cognitive processing (Song et al., 2017). The direction of association between EF and internalising and externalising behaviours, in particular during adolescence, remains therefore unknown.

During adolescence there are structural (Gogtay et al., 2004; Mills et al., 2016; Zhou et al., 2015) and functional (Crone & Dahl, 2012) changes in the frontal-parietal executive networks, structural (Goddings et al., 2014) and functional (Hare et al., 2008) changes in subcortical areas processing emotional stimuli, as well as changes in patterns of connectivity between these networks during emotional regulation (Casey et al., 2019). These changes are thought to lead to increased emotional reactivity during adolescence (e.g. Hare et al., 2008), before the emergence of mature emotional regulation supported by top-down connectivity (i) within the prefrontal cortex and (ii) from the prefrontal cortex to the ventral striatum and amygdala (Casey et al., 2019). Furthermore, the “flexible” state of the PFC has been suggested as a risk factor for adolescent mental health problems (Crone & Dahl, 2012). Neuroimaging studies in adults have shown an association between better down-regulation of emotions, measured behaviourally, and a greater inverse functional connectivity between the amygdala and parts of the prefrontal cortex during emotion regulation (Frank et al., 2014; Lee et al., 2012). Adults with high levels of anxiety have been found to have decreased structural connectivity between prefrontal areas and limbic regions (Kim et al., 2011; Kim & Whalen, 2009). Since the prefrontal cortex is the key brain structure for performing executive functions (Funahashi & Andreau, 2013), it has been suggested that poor EF might contribute to poor emotional regulation via poor top-down regulation of subcortical regions (Frank et al., 2014; White et al., 2011; Zelazo & Cunningham, 2007). Furthermore, cross-sectional clinical and non-clinical studies have found deficits in almost all neuropsychological EF tasks across the spectrum of mental health disorders (Snyder et al., 2015).

Executive functions are a set of cognitive processes necessary for the voluntary control of behaviour and the successful achievement of goals (Diamond, 2013). Although there is no clear agreement as to what executive functions are, they are closely related to fluid intelligence and processing speed (Jurado & Rosselli, 2007). The current dominant adult EF framework (Friedman & Miyake, 2017; Miyake et al., 2000) has focused on three aspects of executive functioning: (i) working memory (WM), the ability to hold and manipulate information in mind; (ii) shifting, the ability to flexibly switch attention between different tasks, rules, or mental states; and (iii) inhibitory control (IC), the ability to suppress distracting information and unwanted responses. Although correlated when measured experimentally, these three aspects of EF are also separable (Karr et al., 2018; Lee et al., 2013; Miyake et al., 2000) and associate distinctly with other measures. For example the updating component of working memory associates more closely with obsessive-compulsive disorder than the other measures, inhibitory control more closely with behavioural disinhibition, and shifting with post-traumatic stress disorder (Snyder et al., 2015; Young et al., 2009). This has led to the proposal of both unity, represented by the “common EF” factor, and diversity, with evidence of unique variance in shifting and updating, and more mixed results of inhibition (Friedman & Miyake, 2017; Karr et al., 2018; Miyake & Friedman, 2012). Developmentally, while results are somewhat mixed, there is a general pattern of increasing specialisation from unity (all EF measures feeding into a common factor) to diversity (different measures tapping into different constructs) (Hartung et al., 2020; Karr et al., 2018). For example, a number of studies consistently find a unitary model of EF in early childhood (Wiebe et al., 2008, 2011), whereas by late childhood/early adolescence the picture is more variable. Some studies find evidence to support three separable but highly correlated traits in 8–13 year-olds (Lehto et al., 2003) and others find that in 5–13 year-olds a two-factor model of EF fits the data best and it is not until age 15 that a three-factor model emerges (Lee et al., 2013). Huizinga et al. (2006) found shifting and WM latent factors in 7–21 year-olds, but no clear IC latent measure, instead the three IC measures loaded separately. Finally, Malagoli and Usai (2018) found a two-factor model when looking at measures of WM and IC in adolescents aged 14–19 years (Malagoli & Usai, 2018).

Early childhood studies find a consistent relationship between poorer EF across a variety of measures and higher levels of externalising behaviours. For example, a meta-analysis of 126 studies including 14,786 participants ranging from preschool to mid-adulthood found a medium effect size for the associations between poorer IC (*d *= 0.56) and WM (*d *= 0.54) and externalising behaviour (Ogilvie et al., 2011). A study of 1,177 6 year-olds found a negative association between IC and measures of the attention and executive functioning domain of the NEPSY-II battery and externalising behaviours (Blanken et al., 2017). Cole and colleagues also found an association in pre-schoolers between a latent EF measure combining IC, WM, shifting, planning and attention tasks, and externalising problems (Cole et al., 1993). Brophy and colleagues, on the other hand, found longitudinal impairments in IC in “hard to manage” children (aged 4 and then 7 years old), but no associations with WM (Brophy et al., 2002). This relationship in younger children between poorer IC and impulsive, intense and aggressive behaviours (Carlson & Wang, 2007; Eisenberg et al., 2001; Eisenberg & Fabes, 1992; Nigg et al., 1999) and hyperactivity (Thorell et al., 2004) is fairly consistent. Beyond early childhood, a study with children and adolescents aged 7–18 years found a strong association between latent measures of externalising and WM (Cassidy, 2016). Séguin et at. (1999) found that the association between aggression and working memory in adolescent boys (13–15 years) remained even after controlling for IQ and ADHD diagnosis (Séguin et al., 1999). In combination, these studies suggest there may be age-related changes to the associations between externalising and EF, where IC is important in early childhood externalising, and WM in later externalising behaviours.

Findings are less consistent regarding associations between EFs and internalising behaviours and disorders, which may be moderated by other factors. For example, pre-schoolers with high inhibitory control are less likely to express negative emotion (Eisenberg & Spinrad, 2004). However, these pre-schoolers also often have poorer cognitive flexibility, which may be behind their greater susceptibility to internalising disorders (Carlson & Wang, 2007; Fox, 1994; Nigg, 2000). Others have found an interaction in pre-schoolers between inhibitory control measured by the day-night inhibitory control task and behavioural inhibition in predicting anxiety (White et al., 2011). Some have argued high levels of IC are protective against developing externalising behaviours, but a risk factor for developing internalising behaviours (Thorell et al., 2004), with suggestions of a quadratic relationship between IC and behavioural problems (Carlson & Wang, 2007). However, clinical studies in adults tend to find a *negative* association between executive functioning and internalising disorders. Poor inhibitory control has been associated with depression as well as rumination in the general population (Hilt et al., 2014; Joormann et al., 2007; Whitmer & Banich, 2007). Depressed adult patients are generally slower and make more errors in inhibitory control tasks (Gohier et al., 2009). Anxiety-related disorders are also associated with visuospatial working memory deficits (Boldrini et al., 2005), while poorer WM has been associated with depression in child and adolescent girls (Matthews et al., 2008) and adults (Harvey et al., 2004). Overall, these results provide a mixed pattern of associations between internalising and EF across ages.

Although the dominant interpretation of the observed associations between cognitive control and emotional behaviours is that poorer EF lead to poorer well-being, there are a number limitations to this interpretation. Current studies are mostly cross-sectional, making it difficult to assess directionality; when longitudinal, the studies mostly fail to account for early correlations between measures or do not allow for emotional behaviours to be the predictive factor in the association (Eisenberg et al., 2009; Hughes & Ensor, 2011). Neuroimaging studies largely use EF tasks with emotional stimuli (Schweizer et al., 2013) and behavioural studies often use self-regulation tasks that are emotionally charged (e.g. Carlson & Wang, 2007), potentially overestimating the correlations between EF and emotion regulation (e.g. see Braunstein et al., 2017). Indeed there are suggestions that EFs recruited in emotional contexts (“hot” EF) operate under different mechanisms to those recruited in unemotional contexts (“cool” EF) (Zelazo & Carlson, 2012). Therefore, evidence is still lacking that “cool” EFs have a causal impact on long-term emotional disorders or problem behaviour.

Neuroimaging studies rarely measure EF directly and use PFC activation as a proxy measure instead; they also leave space for alternative interpretations of the data (for review: Ochsner & Gross, 2008). For example, emotional processing is known to interfere with cognitive processing (Ansari & Derakshan, 2010; Song et al., 2017), therefore it could be that a more reactive amygdala impedes PFC top-down control rather than poor top-down cognitive control on amygdala function resulting in high emotional reactivity. Indeed, Kopf and colleagues performed a neuroimaging study using the emotional N-back and found that WM and emotional processing compete for cognitive resources. Furthermore, in the context of negative valence, emotional content took precedence by limiting brain activation otherwise associated with WM performance (Kopf et al., 2013). Evidence from EF training studies aimed at improving emotional symptoms remain inconclusive (Course-Choi et al., 2017; Hotton et al., 2018; Sari et al., 2016), which also argues against a simple causal link between poor cognitive control and high emotional behaviours.

The alternatives are that, first, both of these models could be true, with different disorders disturbing cognitive control or emotional response networks differently (Goschke, 2014). Second, there could be a reciprocal relationship between EF and emotion. Early development researchers suggest a bi-directional relationship between EF and emotional well-being unfolds over development. For example, infant orienting away from distressing stimuli establishes early self-regulation and cognitive control, setting a positive foundation for working memory, which results in better emotion regulation and in turn frees up cognitive space for better cognitive development (Bell & Wolfe, 2004; Posner & Rothbart, 2000).

There remains significant work to do to better understand the direction of associations between cognitive control and emotional behaviours. Despite theories suggesting a particular vulnerability in adolescence due to changes in the neural circuity underpinning emotion regulation (Mills et al., 2014; Prencipe et al., 2011) little research has been done into how cognitive control, emotional regulation and emotional behaviours relate to each other and change over adolescence. The present study used a longitudinal adolescent dataset to investigate the direction of associations between IC and WM measures of EF and parent-report measures of internalising and externalising emotional behaviours. The focus was on the spectrum of internalising and externalising behaviours, rather than on extreme clinical ends of the distributions. While a few studies have used latent factors of multiple EF measures (e.g. Cassidy, 2016; Cole et al., 1993), much research in this field has used only one task per construct and non-latent scores (e.g. Brophy et al., 2002; Thorell et al., 2004). The dominant adult EF framework argues latent measures are necessary to remove task-specific noise from EF tasks (Friedman & Miyake, 2017; Miyake & Friedman, 2012; Snyder et al., 2015). We therefore created two models: one which included IC and WM mean scores, the other using confirmatory factor analysis to create latent scores. As reviewed above, EF can be modelled as a single latent factor or multiple factors. We therefore compared a model of EF with two separate factors representing WM and IC, and a unitary EF factor model, and used the better model in the subsequent latent cross-lagged panel analysis. The aim was not to make specific claims about the structure of EF as we lacked a shifting measure and multiple measures of each construct, instead we aimed to assess whether the directionality of longitudinal associations between EF and externalising and internalising remained consistent across models.

Measures of executive function were collected in the Avon Longitudinal Study of Parents And Children (ALSPAC) cohort during early adolescence (age ∼10 years) and mid- to late adolescence (age ∼15–17 years), therefore this was the time interval that was considered here. There is some evidence to show that mid-adolescence is a period of particular emotional turmoil (see Ahmed et al., 2015), which this study will not be able to capture. However, the advantages of these time points are that the early measures allow us to take a “baseline” prior to the main developmental changes of adolescence, and the later measures allow us to capture long-lasting effects that would be relevant for long-term mental health issues. Importantly, the EF measures used in the present study did not involve emotional stimuli, which means any observed associations between EF and emotional behaviours would not be driven by the involvement of emotion regulation in the EF tasks.

We constructed cross-lagged panel models to assess firstly whether there was an association between cognitive control and emotional behaviours during adolescence and whether these associations continued across development. Secondly, we assessed whether cognitive control or emotional behaviours were the drivers of change over time. We expected a reciprocal developmental explanation where better mental health allows for better cognitive development and vice versa. Therefore, we expected that working memory and inhibitory control and internalising and externalising would have bi-directional relationships over adolescence.

## Method

### Study cohort

The Avon Longitudinal Study of Parents And Children (http://www.bristol.ac.uk/alspac/) is an on-going population-based study investigating factors influencing development and health. Initial recruitment included 14,541 mothers with 13,988 children alive at age one. Another round of recruitment at around age 7 left the total sample size for data collected after this age at 15,247 (see Supplementary Note 1 and Boyd et al., 2013 for full details). The study website contains details of all the data that are available through a fully searchable data dictionary (http://www.bris.ac.uk/alspac/researchers/data-access/data-dictionary/). Inevitably not all participants attended all data collection waves or returned all questionnaires, and therefore the final sample for the current study includes 1,445 participants (622 males, 823 females) aged 10 years 3 months to 13 years 3 months in the early measures and 14 years 3 months to 19 years 6 months in the late measures, who provided data for all measures at both time-points (see Supplementary Note 1 and Supplementary Table 1 for a description of the full and final samples). Compared to the full cohort, the final sample had a significantly greater proportion of females, was overall of higher socio-economic status, IQ, working memory and early inhibitory control, and reported fewer internalising and externalising behaviours, but with very small effect sizes (Supplementary Table 1). A post-hoc power analysis for our main analyses using a multiple regression model with four predictors showed we had a power of 1 to detect a R^2^ of 0.02. Ethical approval for the study was obtained from the ALSPAC Ethics and Law Committee and the local research Ethics Committee.

### Measures

#### Strengths and difficulties questionnaire

Internalising and externalising measures were collected using the Strengths and Difficulties Questionnaire (SDQ) (Goodman, 1997), completed by a parent when the participant was 11 and 17 years old. ALSPAC did not have child-report measure of the SDQ before 16 years old and therefore for consistency we used parent-report measures at both timepoints. The SDQ is a well-validated measure of childhood behavioural and mental health problems (Ford et al., 2007; Goodman et al., 2010), including the parent-report measure specifically (Becker et al., 2004a). There is reasonable consistency between parent-report and self-report measures across this age group (Goodman et al., 1998) and parent-report measures are a better predictor of clinical diagnosis in younger adolescents than self-report measures (Becker et al., 2004b). The internalising measure comprises the five questions from the emotional problems scale (e.g. “Often unhappy, downhearted”) and the five questions from the peer problems scale (e.g. “Rather solitary, tends to play alone”). The externalising measure comprises the five questions from the conduct problems scale (e.g. “Often has temper tantrums or hot tempers”) and the five questions from the hyperactivity scale (e.g. “Restless, overactive”). For each question, the respondent can choose the answers “Not true”, “Somewhat True” or “Certainly True”, which have a score of 0, 1 and 2 respectively (note reversed-scored items were recoded). Sum scores resulted in possible scores of 0–20 for each scale at each time point. The internalising-externalising scales of the SDQ have been found to be good predictors of mental health issues in the general population (Goodman et al., 2010).

#### Early executive function measures

Participants performed two EF tasks at age 10 years: the Counting Span task and the Stop Signal task. The Counting Span task (Case et al., 1982) is a WM task where at the end of each block of trials the participant is asked to recall in order the number of red dots presented on each trial of that block. A *Counting Span score* was calculated from the number of trials correctly recalled (max. 42) (Table 1). The Stop Signal task (Logan & Cowan, 1984) is an IC task where the participant must respond to X’s and O’s on the screen by pressing the corresponding button as quickly as possible (Go trials). In a first practice blocks participants complete Go trials. This establishes a mean baseline reaction time (RT). The second practice block introduces Stop trials. On Stop trials a beep played randomly 150 ms or 250 ms before the participant’s baseline RT indicates the participant should refrain from responding. The practice block included 16 Go trials and eight Stop trials. There were then two experimental blocks of 48 trials, 16 of which were Stop trials (33%). As the number of correct Stop trials in the 150 and 250 ms delay conditions were highly correlated, an average *Stop Signal number of correct Stop trials* across delays was calculated for each individual (Table 1).

**Table 1.** Descriptive statistics of individual measures in the complete case dataset (*n* = 1,445).

Measure	Age at testing	*M* (range)	*SD*
Early internalising^a^ *[SDQ peer + emotional problems]*	11y8m–13y3m	2.1 (0–15)	2.4
Late internalising^a^ *[SDQ peer + emotional problems]*	16y6m–18y4m	2.1 (0–17)	2.4
Early externalising^a^ *[SDQ conduct + hyperactivity problems]*	11y8m–13y3m	3.1 (0–19)	2.6
Late externalising^a^ *[SDQ conduct + hyperactivity problems]*	16y6m–18y4m	2.8 (0–18)	2.6
Early working memory *[Counting Span score]*	10y3m–11y11m	19.9 (0–42)	7.7
Late working memory *[2-back accuracy] (%)*	16y3m–19y6m	80.6 (5–100)	19.8
Early inhibitory control *[Stop Signal N correct Stop trials]*	10y3m–11y11m	12.9 (0.5–16.0)	2.6
Late inhibitory control *[Stop Signal N correct Stop trials]*	14y3m–17y1m	0.0 (−13.6–2.6)^b^	2.2

^a^ Sum scores.

^b^ This score represents the residuals saved after regressing out the different task parameters used for different groups of participants.

SDQ: Strengths and Difficulties Questionnaire.

#### Late executive function measures

The Counting Span task was not repeated in later testing sessions within the ALPSAC study, however an N-back task was used at age 17 years. This is another standard WM test, although rather than assessing a WM span it requires updating items in WM. Participants were presented with numbers 0–9 for 500 ms and had 3000 ms to judge whether the current number was the same as the number shown either two or three trials before (2-back or 3-back). The practice block consisted of 12 trials with two targets, and there were single blocks of the 2-back and 3-back conditions each consisting of 48 trials with eight targets. The measure used from this task was the *2-back accuracy* as it has also been used in other studies looking at the relationship between WM and psychopathology and is the most commonly used measure (Snyder et al., 2015). The Stop Signal task from age 10 was repeated at age 15 years. However, although the practice and test blocks at age 15 were the same as at age 10, delay times between stimulus and stop signal presentations were different for different groups of participants. For this reason, a residual score covarying for the differences in delay duration was calculated for the purpose of this study. Latent measures of early and late EF were created using the saved factor scores from a confirmatory factor analysis.

Although shifting is another key construct of executive function, there was no available measure of shifting during adolescence in the ALSPAC sample.

### Statistical analysis

We used the Lavaan version 0.6–7 (Rosseel, 2012) structural equation modelling package in R (R Core Team, 2013) with Robust Maximum Likelihood estimator with Yuan-Bentler scaled statistic (MLR) to account for any violation of multivariate normality. Due to the fact that the measures within each time point were collected at slightly different ages (see Table 1) and that there are sex differences on some measures (Donati et al., 2019) all EF and SDQ scores were regressed for age and sex at each time point. Confirmatory factor analyses (CFA) were performed on EF measures to create latent scores using complete case data (*n* = 1,445) (see Supplementary Figure 1) in order to assess whether a single EF model was a better fit than a model with separate WM and IC latent measures, although we note that a single task was available at each timepoint for each EF construct. Next, two cross-lagged panel structural equation models were used to investigate the longitudinal bi-directional associations between (i) the WM and IC executive function measures and the internalising and externalising sum scores and (ii) the latent executive function measure/s and internalising and externalising sum scores. A panel model postulates that there is a directional relationship between constructs using regression. Cross-sectional correlations between measures at the early time point were controlled for in order to understand effects specific to changes between the time points.

## Results

Pearson’s correlations were calculated between the eight variables included in the cross-lagged model (see Supplementary Table 2). Early and late internalising and externalising behaviours were positively correlated [range: .22–.59]. Weaker correlations were observed between early and late working memory and inhibitory control [range: .08–.51]. Parent-reported problem behaviours and EF cognitive measures were negatively correlated [range: -.06–-.20], with lower correlations between internalising and EF measures than between externalising and EF measures.

**Figure 1.**
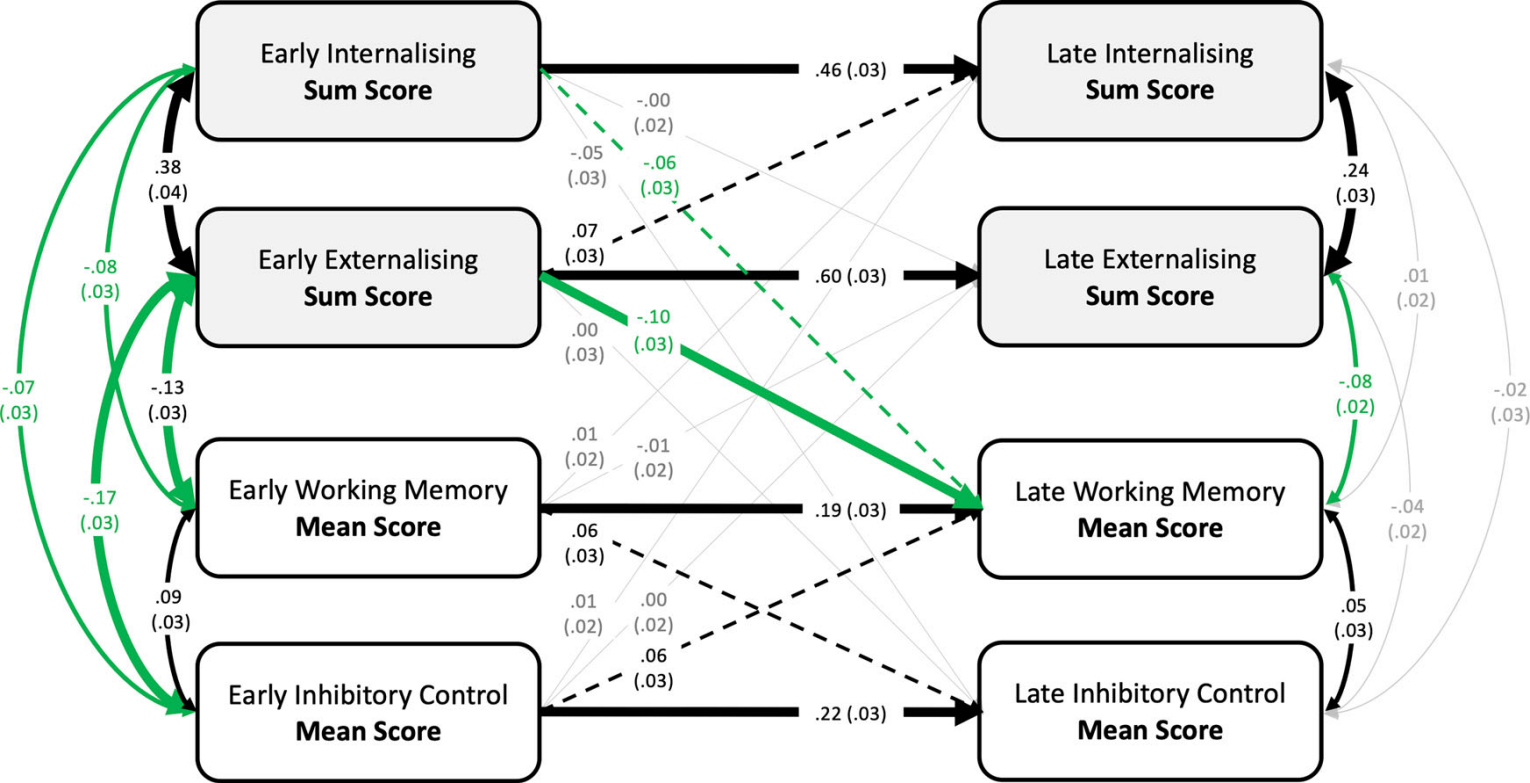
Cross-lagged panel model of the associations between working memory and inhibitory control and parent-reported internalising and externalising behaviours in early and mid-to-late adolescence. Values represent standardised betas with standard errors in brackets. Line styles indicate significance: thick lines *p* ≤ 001, thin lines *p* ≤ .01, dashed lines *p* ≤ .05, grey lines *p* > .05. Green lines highlight significant cross-construct associations. Age at testing: Early = 10y3m–13y3m, Late = 14y3m–18y4m.

The cross-lagged panel model 1 is shown in Figure 1. In combination, predictors explained the following percentage of variance in the late measures: 6.9% for working memory, 6.1% for inhibitory control, 24.2% for internalising, and 35.3% for externalising.

When controlling for all associations in early adolescence, all four measures were significantly (*p* ≤ .01) correlated with each other in early adolescence (Figure 1). In late adolescence, when previous associations were controlled for, only three associations remained significant (internalising – externalising, externalising – WM, and WM – IC, Figure 1).

Early internalising longitudinally predicted variance in late internalising and late working memory. Similarly, early externalising longitudinally predicted variance in late externalising and late working memory, and in addition predicted variance in late internalising. The strongest cross-construct association was between early externalising and late working memory. Early EF measures predicted variance in late adolescence EF measures but did not predict variance in internalising or externalising in late adolescence (Figure 1).

Confirmatory factor models comparing one and two EF factors found the two factor model returned a significantly better model fit χ^2^(9) = 1985.727, *p* < .001; CFI = .997; RMSEA = .020 [.000, .039]; SRMR = .012, χ^2^*_diff _*= 173.61*,* df*_diff _*= 4*, p* < .001. Therefore, even though measures from only a single task was available for each EF construct, the model with two EF factors was used in the subsequent cross-lagged panel model. In the latent EF trait cross-lagged panel analysis (Supplementary Figure 1) significant relationships remained the same but were strengthened between internalising and working memory and within EF measures. The variance explained in the late measures by all predictors for model 2 was 33.1% for WM, 11.0% for IC, 24.2% for internalising and 35.3% for externalising.

## Discussion

The present study demonstrated that executive functions and internalising and externalising behaviours showed significant cross-sectional correlations during early adolescence, replicating previous findings. However, when controlling for early associations, analyses showed that internalising and externalising in early adolescence predicted later executive functioning, while early executive function measures did not predict later internalising or externalising. Strengths of this study are the use a of large population-based longitudinal sample, the comparison of different models and the consideration of bi-directional associations between emotional behaviours and EF.

Internalising and externalising were significantly cross-sectionally correlated with both WM and IC in early adolescence, in line with previous findings in childhood (e.g. Carlson & Wang, 2007), adolescence (e.g. Ogilvie et al., 2011) and adulthood (e.g. Snyder et al., 2015). All the associations were found to be negative. This is contrary to the preschool children results that suggest that higher IC associates with higher internalising (White et al., 2011), indicating that by adolescence this pattern is not present or has changed. The negative associations were overall small. The first cross-lagged panel model showed that when controlling for early associations, the only cross-construct cross-sectional correlation that remained significant in late adolescence was between WM and externalising, suggesting an increase in association between the two constructs over the course of adolescence, or that WM and externalising are similarly influenced by individual differences in some other aspect of cognition or the environment over the course of adolescence. These results replicate previous findings of associations between externalising behaviours and working memory from pre-schoolers to adults (Ogilvie et al., 2011; Ziermans et al., 2012), and between inhibitory control and externalising in childhood (Carlson & Wang, 2007; Eisenberg & Fabes, 1992) but suggest that findings linking externalising behaviour and IC in adulthood (Li et al., 2006) may represent residual relationships established early in life or common variance shared with working memory.

The high level of cross-sectional and longitudinal correlations between internalising and externalising, as has been shown previously, suggests a strong relationship between the two constructs that continues to change over time. The model shows that even when controlling for their association in early adolescence, the associations between internalising and externalising in late adolescence remains large. Studies spanning from infancy to adolescence suggest that externalising difficulties peak early in childhood whereas internalising difficulties continue to increase steadily with age and these changes are attributed to cognitive development (Gilliom & Shaw, 2004). In the present sample we find that early externalising is associated with later internalising, suggesting a possible change in the expression of emotional problems.

While EF and emotional behaviours show small cross-sectional associations, EF was not found to predict later internalising or externalising behaviours. This indicates that, when controlling for childhood associations, executive functions are not directly influencing change in emotional behaviours during adolescence, contrary to what is predicted by Gilliom and Shaw (2004) and to the widely held view that individual differences in EF influence changes in emotional behaviours over adolescence (Ahmed et al., 2015). It is possible that EF’s influence on the emergence of emotional behaviours may be limited to childhood. Hughes and Ensor (2011) for example, find that change in EF between 4 and 6 years of age predicts internalising and externalising at 6 years. However, while many studies have modelled executive function over development (Huizinga et al., 2006; Lee et al., 2013; Malagoli & Usai, 2018), and some studies have looked at whether executive function can predict emotional behaviours over development (Hughes & Ensor, 2011), we are aware of only one other study that models bi-directional effects of emotional behaviour on executive function across development as was done in the present study. Brieant and colleagues collected WM, IC and shifting measures as well as internalising and externalising at four time-points across adolescence, beginning at 13–14 years of age. They allowed for bi-directional effects across adolescence and found the same results as we observed in the present study: while EF did not predict changes in internalising or externalising, both measures of emotional behaviours predicted changes in EF (Brieant et al., 2020). Furthermore, they covered the period of adolescence missed in the present study (13–17 years old), suggesting our results are unlikely to be due to missing a key period. Combined, the present study and the study by Brieant and colleagues (Brieant et al., 2020) provide evidence that problem behaviours in early to mid-adolescence predict the longitudinal development of executive functioning into late adolescence, rather than the other way around.

There is evidence showing that adaptive emotion regulation strategies correlate with better EF and emotional well-being, as well as increased PFC and reduced amygdala activation during emotion regulation tasks (Ochsner et al., 2012). As the present study found that both early internalising and externalising behaviours predicted later executive function, unsuccessful top-down control could therefore be a consequence rather than a cause of increased emotionality. This interpretation aligns with studies that document how emotional processing interferes with cognitive processing (Fuhrmann et al., 2019; Song et al., 2017). Studies of emotion regulation and emotion interference generally focus on state interactions between emotion and cognition, whereas we have used more trait-like measures. A possible integrative interpretation of the results of previous research and the present study is that top-down executive control is involved in regulating state emotion but does not alter trait emotionality in adolescence. Alternatively, there could be a bi-directional relationship, as posited by Bell and Wolfe (2004), where abilities are dominant at different times in development. EF may be dominant in influencing the early development of temperament when EFs are developing at a fast rate. However, by adolescence, although EFs are still flexible, they are potentially more affected by fluctuating emotional states that are driven by increased reward-salience, sensation seeking and salience of social interactions (Blakemore & Robbins, 2012; Crone & Dahl, 2012; Steinberg et al., 2018).

Our comparison of models specifying one or two EF factors showed a two-factor model was a significantly better fit. It is possible that this reflects the hypothesised increased specialisation and differentiation of execution functions over development (Karr et al., 2018). The cross-lagged panel model using latent EF variables (Supplementary Figure 1) showed the same patterns of associations as the basic model, but with slightly stronger associations between the EF measures.

Limitations

Overall the associations observed across constructs were small (although not unexpected given the time gap and all the variables controlled for) and a number of limitations due to the use of an existing dataset should be considered.

A first set of limitations relates to the cognitive tasks. Different WM tasks were administered to ALSPAC participants in early and late adolescence. It is therefore possible that the correlations between WM and internalising and externalising in early adolescence may not be fully controlled for. The Stop Signal task measure used in ALSPAC only included two Stop Signal delays rather than the variable delays typically used to assess Stop Signal Reaction Times (Logan & Cowan, 1984) and unfortunately, no measure of cognitive flexibility was available. The latent model was restricted by the fact that each factor had only variables from one task, which reduces the traditional advantages of using latent variables to remove task-specific variance. Finally, only a small amount of variance in late WM and IC was explained by model 1 (6.9% and 6.1% respectively), which perhaps reflects the commonly reported low level of test re-test reliability of EF measures (Hedge et al., 2018). Although this was considerably larger in the latent EF model: 33.1% for WM and 11% for IC.

Another set of limitations relates to the age of the participants and the nature of the sample. The ages at which participants completed the EF tasks and their parents completed the SDQ were not exactly matched, which may have affected the results. Overall, the size of the longitudinal associations between EF and emotional behaviours were small, perhaps due to the use of a combination of experimental and questionnaires measures but also the fact that this was a sample with generally low behaviour problems and high EF. Although our subsample was taken from a population-based study, the subsample had significantly higher levels of cognitive ability and lower levels of internalising and externalising than the main cohort. It is not clear how this would have influenced the results, but it may explain the relatively smaller effect sizes found here rather than in clinical studies (Snyder et al., 2015). It is also possible that the current study misses out on interactions between EF and emotional behaviour changes occurring in mid-adolescence. Another set of measures taken in mid-adolescence would have allowed us to track the continuing evolution of this relationship more closely, however the study by Brieant et al. (2020) suggests it might not have altered our findings. A further possibility is that the effect sizes are small in adolescence and continue to get smaller with age, as brain functions continue to differentiate and specialise and individual differences in cognitive processes become more differentiated.

Finally, we note that although the SDQ is widely used and has shown fairly good reliability in predicting internalising disorders (Becker et al., 2004a; Goodman et al., 2010), the internalising measure of the SDQ may be too focused on depression and the peer interaction items may reflect individual differences unrelated to internalising (e.g. “rather solitary” may reflect an autism spectrum disorder or the consequences of externalising behaviours); it is possible that elements not captured are more closely related to EF. Self-report rather than parent-report measures of mental health may have given us a different picture of the relationship than that what we would possibly gain from self-report. However, assessments of parent-report measures tend to find them accurate (Becker et al., 2004a; Goodman et al., 1998), although sometimes better at reporting on externalising rather than internalising behaviours (Becker et al., 2004b).

## Conclusion

This study replicated the widely reported cross-sectional associations between EF and internalising and externalising behaviours. However, the longitudinal design demonstrated that when controlling for childhood associations, externalising, and to a lesser extent, internalising, in early adolescence is associated with later executive functioning, but not the other way around. These results suggest that while executive function training in early adolescence may not improve emotional well-being, interventions to improve well-being may have a positive influence on cognitive performance. Further studies could incorporate middle adolescence as well as charting growth trajectories.

## Supplementary Material

Supplementary_Material
