# Supplementary material for: Internalising and externalising in early adolescence predict later executive function, not the other way around: a cross-lagged panel analysis
Source: Cogn Emot. 2021 Apr 26;35(5):986–98. doi: 10.1080/02699931.2021.1918644 (PMC8372297; doi:10.1080/02699931.2021.1918644)
Supplement: Supplementary_Material [file PCEM_A_1918644_SM1654.docx]

**Supplementary material**

**Supplementary Note 1: Description of study cohort numbers**

Pregnant women resident in Avon, UK with expected dates of delivery 1st April 1991 to 31st December 1992 were invited to take part in the study. The initial number of pregnancies enrolled is 14,541 (for these at least one questionnaire has been returned or a “Children in Focus” clinic had been attended by 19/07/99). Of these initial pregnancies, there was a total of 14,676 foetuses, resulting in 14,062 live births and 13,988 children who were alive at 1 year of age.

When the oldest children were approximately 7 years of age, an attempt was made to bolster the initial sample with eligible cases who had failed to join the study originally. As a result, when considering variables collected from the age of seven onwards (and potentially abstracted from obstetric notes) there are data available for more than the 14,541 pregnancies mentioned above.

The number of **new pregnancies** not in the initial sample (known as Phase I enrolment) that are currently represented on the built files and reflecting enrolment status at the age of 18 is 706 (452 and 254 recruited during Phases II and III respectively), resulting in an additional 713 children being enrolled. The phases of enrolment are described in more detail in the cohort profile paper (Boyd et al., 2013).

The total sample size for analyses using any data collected after the age of seven is therefore 15,247 pregnancies, resulting in 15,458 foetuses. Of this **total sample** of 15,458 foetuses, 14,775 were **live births** and 14,701 were **alive at 1 year of age**. For full cohort data see Boyd et al. (2013).

**Supplementary Table 1:** Demographic and summary data for the entire ALSPAC sample and the sub-sample used in this study and statistical results of comparisons of the two samples.

|  |  | Gender (%males) | SES^a^ | Verbal IQ^b^ | Matrix IQ^b^ | Early Int | Early Ext | Late Int | Late Ext | Early WM | Early IC | Late WM | Late IC |
| --- | --- | --- | --- | --- | --- | --- | --- | --- | --- | --- | --- | --- | --- |
| Full Data | Range |  | 1 - 6 | 4 - 71 | 2 - 80 | 0 - 17 | 0 - 20 | 0 - 18 | 0 - 18 | 0 - 42 | 0 - 16 | 0.05 - 1 | -14.11 -2.57 |
| *N* = 15,445 | Mean | 51.40 | 3.95 | 45.42 | 24.68 | 2.42 | 3.83 | 2.40 | 3.38 | 18.52 | 12.83 | 0.78 | 0^c^ |
|  | Missing | 591 | 3,788 | 10,165 | 10,169 | 8,882 | 8,738 | 10,535 | 10,396 | 8,439 | 8,476 | 11,851 | 10,194 |
| Paper Data | Range |  | 1 - 6 | 17 - 70 | 6 - 80 | 0 - 15 | 0 - 19 | 0 - 17 | 0 - 18 | 0 - 42 | 0.5 - 16 | 0.05 - 1 | -13.61 -2.57 |
| *n* = 1,445 | Mean | 43.05 | 4.31 | 47.71 | 25.41 | 2.11 | 3.09 | 2.10 | 2.79 | 19.87 | 12.95 | 0.81 | 0.02 |
|  | Missing | 0 | 42 | 11 | 13 | 0 | 0 | 0 | 0 | 0 | 0 | 0 | 0 |
| Comparison of the two samples | | *X*^2^(1) = 36.78, *p* < .001, Cramer’s *V* = 0.05 | *t*(1856) = 13.35,  *p* < .001, Hedge’s *g*_s_ = .11 | *t*(2411) = 8.10,  *p* < .001, Hedge’s *g*_s_ = . 07 | *t*(2416) = 3.65,  *p* < .001, Hedge’s *g*_s_ = . 03 | *t*(2237) = 4.29,  *p* < .001, Hedge’s *g*_s_ = . 04 | *t*(2409) = 9.55,  *p* < .001, Hedge’s *g*_s_ = . 08 | *t*(2547) = 4.32,  *p* < .001, Hedge’s g_s_ = . 04 | *t*(2544) = 7.43,  *p* < .001, Hedge’s *g*_s_ = .06 | *t*(2066) = 6.04,  *p* < .001, Hedge’s *g*_s_ = .05 | *t*(2153) = 1.52,  *p* < .001, Hedge’s *g*_s_ = .01 | *t*(2764) = 3.52,  *p* < .001, Hedge’s *g*_s_ = .03 | *t*(2335) = 0.25,  *p* = .804, Hedge’s *g*_s_ = .00 |

^a^ Socioeconomic status (SES) was calculated using the mother and partner's occupational social classes as classified by the Office of Population Census and Surveys (OPCS) (i.e., 6 = professional, 1 = unskilled manual labour; OPCS, 1990). For each individual, an average score was created from the parental scores averaged across three times points: the first taken before the birth of the child (mother), then when the child was 4 years of age (mother and partner) and 8 years of age (partner).

^b^ The Vocabulary and Matrix Reasoning WASI raw scores were taken from the Wechsler Abbreviated Scale of Intelligence (WASI, Wechsler, 1999) interview performed at age 15.

Ext: externalising; IC: inhibitory control; Int: internalising; WM: working memory.

^c^ These are saved residuals and therefore the mean is always 0.

**Supplementary Table 2**: Pearson’s correlations coefficients (*r*) between executive function and internalising and externalising raw and latent variables. Higher scores in internalising (Int) and externalising (Ext) indicate more problem behaviours, while higher scores in working memory (WM) and inhibitory control (IC) indicate better executive functioning (EF). Gender and age have been regressed out of all measures. Cross-construct correlations are highlighted in bold.

|  |  | Behavioural problems | | | | Raw EF Scores | | | | Latent EF Scores | | |
| --- | --- | --- | --- | --- | --- | --- | --- | --- | --- | --- | --- | --- |
|  |  | Early Int | Early Ext | Late Int | Late Ext | Early WM | Early IC | Late WM | Late IC | Early WM | Early IC | Late WM |
| Behavioural Problems | Early Ext | 0.381^***^ |  |  |  |  |  |  |  |  |  |  |
|  | Late Int | 0.487^***^ | 0.247^***^ |  |  |  |  |  |  |  |  |  |
|  | Late Ext | 0.224^***^ | 0.594^***^ | 0.314^***^ |  |  |  |  |  |  |  |  |
| Raw EF Scores | Early WM | **-0.078^**^** | **-0.134^***^** | -0.039 | -0.089^***^ |  |  |  |  |  |  |  |
|  | Early IC | **-0.073^**^** | **-0.174^***^** | -0.036 | -0.102^***^ | 0.090^***^ |  |  |  |  |  |  |
|  | Late WM | -0.113^***^ | -0.155^***^ | **-0.057^*^** | **-0.154^***^** | 0.216^***^ | 0.094^***^ |  |  |  |  |  |
|  | Late IC | -0.074^**^ | -0.069^**^ | **-0.055^*^** | **-0.072^**^** | 0.083^**^ | 0.232^***^ | 0.084^***^ |  |  |  |  |
| Latent EF Scores | Early WM | -0.078^**^ | -0.134^***^ | -0.039 | -0.089^***^ | 1.000^***^ | 0.090^***^ | 0.216^***^ | 0.083^**^ |  |  |  |
|  | Early IC | -0.073^**^ | -0.178^***^ | -0.043 | -0.101^***^ | 0.105^***^ | 0.965^***^ | 0.117^***^ | 0.258^***^ | 0.105^***^ |  |  |
|  | Late WM | -0.144^***^ | -0.201^***^ | -0.06^*^ | -0.178^***^ | 0.507^***^ | 0.266^***^ | 0.805^***^ | 0.242^***^ | 0.507^***^ | 0.301^***^ |  |
|  | Late IC | -0.063^**^ | -0.069^**^ | -0.052^*^ | -0.075^**^ | 0.115^***^ | 0.283^***^ | 0.110^***^ | 0.936^***^ | 0.115^***^ | 0.319^***^ | 0.296^***^ |

^***^ *p*$\leq$ .001, ^**^ *p*$\leq$ .01, ^*^ *p* $\leq$ .05


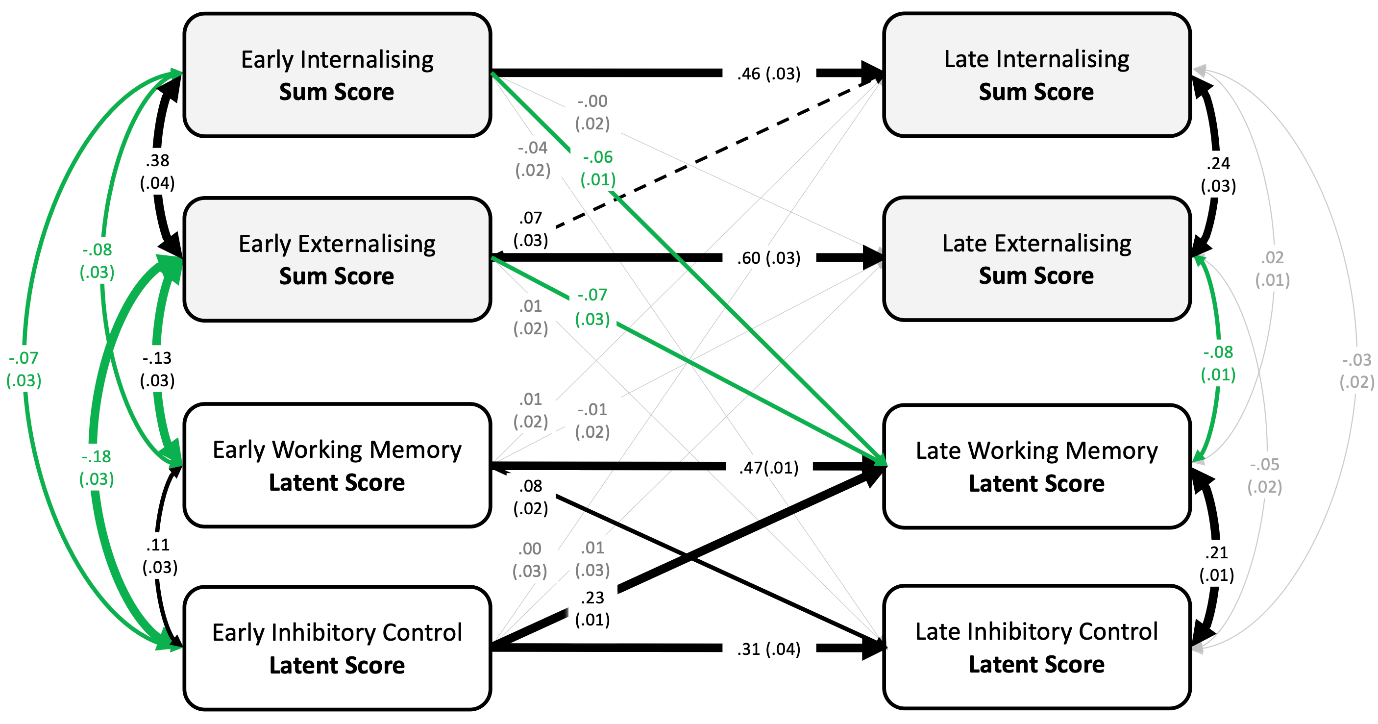


**Supplementary Figure 1:** Cross-lagged panel model of the associations between latent working memory and inhibitory control and parent-reported sum scores of internalising and externalising problem behaviours in early and mid-to-late adolescence. Values represent standardised betas with standard errors in brackets. Line styles indicate significance: thick lines *p* $\leq$ .001, thin lines *p* $\leq$ .01, dashed lines *p* $\leq$ .05, grey lines *p* > .05. Green lines highlight significant cross-construct associations. Age at testing: Early = 10y3m - 13y3m, Late = 14y3m - 18y4m

**Supplementary references**

Boyd, A., Golding, J., Macleod, J., Lawlor, D. A., Fraser, A., Henderson, J., ... & Davey Smith, G. (2013). Cohort profile: the ‘children of the 90s’—the index offspring of the Avon Longitudinal Study of Parents and Children. *International journal of epidemiology*, *42*(1), 111-127.

Hu, L., & Bentler, P. M. (1999). Cut-off criteria for fit indexes in covariance structure analysis: Conventional criteria versus new alternatives. *Structural Equation Modelling: A Multidisciplinary Journal*, *6*(1), 1–55.

Schreiber, J. B., Nora, A., Stage, F. K., Barlow, E. A., & King, J. (2006). Reporting Structural Equation Modelling and Confirmatory Factor Analysis Results: A Review. *The Journal of Educational Research*, *99*(6), 323–338.

Wechsler, D. (1999). Wechsler Abbreviated Scale of Intelligence. The Psychological Corporation: Harcourt Brace & Company. New York, NY.
